# Vaccinate-assess-move method of mass canine rabies vaccination utilising mobile technology data collection in Ranchi, India

**DOI:** 10.1186/s12879-015-1320-2

**Published:** 2015-12-29

**Authors:** Andrew D. Gibson, Praveen Ohal, Kate Shervell, Ian G. Handel, Barend M. Bronsvoort, Richard J. Mellanby, Luke Gamble

**Affiliations:** Mission Rabies, 4 Castle Street, Cranborne, BH21 5PZ Dorest, UK; HOPE & Animal Trust, 21/1 Mandir Marg, Birsa Nagar, P O Hatia, Ranchi, 834003 Jharkhand India; Royal (Dick) School of Veterinary Studies, Division of Veterinary Clinical Studies, The University of Edinburgh, Hospital for Small Animals, Easter Bush Veterinary Centre, Roslin, Midlothian, EH25 9RG UK; The Roslin Institute at the Royal (Dick) School of Veterinary Studies, Division of Genetics and Genomics, The University of Edinburgh, Hospital for Small Animals, Easter Bush Veterinary Centre, Roslin, Midlothian, EH25 9RG UK

**Keywords:** Rabies, Vaccination, Mark, Capture, Dog, Population

## Abstract

**Background:**

Over 20 000 people die from rabies each year in India. At least 95 % of people contract rabies from an infected dog. Annual vaccination of over 70 % of the dog population has eliminated both canine and human rabies in many countries. Despite having the highest burden of rabies in the world, there have been very few studies which have reported the successful, large scale vaccination of dogs in India. Furthermore, many Indian canine rabies vaccination programmes have not achieved high vaccine coverage.

**Methods:**

In this study, we utilised a catch-vaccinate-release approach in a canine rabies vaccination programme in 18 wards in Ranchi, India. Following vaccination, surveys of the number of marked, vaccinated and unmarked, unvaccinated dogs were undertaken. A bespoke smartphone ‘Mission Rabies’ application was developed to facilitate data entry and team management. This enabled GPS capture of the location of all vaccinated dogs and dogs sighted on post vaccination surveys. In areas where coverage was below 70 %, catching teams were re-deployed to vaccinate more dogs followed by repeat survey.

**Results:**

During the initial vaccination cycle, 6593 dogs were vaccinated. Vaccination coverage was over 70 % in 14 of the 18 wards. A second cycle of vaccination was performed in the 4 wards where initial vaccination coverage was below 70 %. Following this second round of vaccination, coverage was reassessed and found to be over 70 % in two wards and only just below 70 % in the final two wards (66.7 % and 68.2 %, respectively).

**Conclusion:**

Our study demonstrated that mobile technology enabled efficient team management and rapid data entry and analysis. The vaccination approach outlined in this study has the potential to facilitate the rapid vaccination of large numbers of dogs at a high coverage in free roaming dog populations in India.

**Electronic supplementary material:**

The online version of this article (doi:10.1186/s12879-015-1320-2) contains supplementary material, which is available to authorized users.

## Background

Rabies is a devastating zoonotic disease which kills an estimated 59,000 people per year [1]. The global cost of rabies has been estimated to be 8.6 billion USD and causes the loss of over 3.7 million disability-adjusted life years [1]. In many developing countries dogs are allowed to roam freely and are the principal reservoir for the disease, with almost all human cases of rabies contracted from the bites of an infected dog [2]. Mass vaccination of the dog population has been shown to be effective at eliminating the disease from many countries [1, 3, 4]. This has led to the broad belief that the global elimination of canine transmitted rabies is possible through mass dog vaccination [5–7]. Despite the feasibility of eliminating both canine and human rabies through widespread canine vaccination programmes, there is still limited investment in large scale dog vaccination approaches in many African and Asian countries where the disease remains endemic [1].

India accounts for over 35 % of the global rabies burden with over 20 000 deaths a year attributed to rabies. Despite the need to develop and undertake mass canine vaccination programmes in India, there are few published reports of successful implementation of large scale vaccination programmes [8, 9]. There is a particular dearth of research relating to the Indian dog population and practical implementation of effective mass canine vaccination on a scale that could be broadened to a state-wide or even national level [10–12]. A major challenge to the eradication of rabies in India is ensuring that not only are a large number of dogs vaccinated, but also that the vaccination coverage is sufficiently high to break the transmission cycle in the dog population. There is a broad consensus that over 70 % of dogs need to be vaccinated in order to have a significant impact on the incidence of rabies in dog and human populations [2]. Central point vaccination campaigns have been effective at accessing a large enough proportion of the dog population to impact on canine and human rabies incidence in parts of Africa [13]. However, these approaches have been ineffective at reaching a high proportion of the Indian dog population where the majority of dogs are free roaming [10].

In order to progress towards the eradication of canine transmitted rabies in India, it is essential that effective field protocols are developed which facilitate mass canine vaccination at sufficiently high vaccination coverage to break the cycle of transmission within the dog population. Firstly, there is a clear need to develop field strategies which allow vaccination of large numbers of free roaming dogs in urban areas. Secondly, there is a need for improved methodologies which can rapidly assess whether the vaccination coverage achieved is high enough to result in widespread protective immunity within the dog population. This study describes the development and implementation of a mass vaccination programme in Ranchi, India which resulted in the vaccination of over 6500 dogs. Crucially, we developed a ‘Mission Rabies’ smartphone application (App) which allowed for rapid entry of field data and facilitated the real time assessment of vaccination coverage. This mobile technology ensured that areas of vaccination coverage below 70 % were immediately detected, thereby enabling areas with suboptimal vaccination coverage to be revisited by vaccination teams.

## Methods

### Study area

**Fig. 1:**
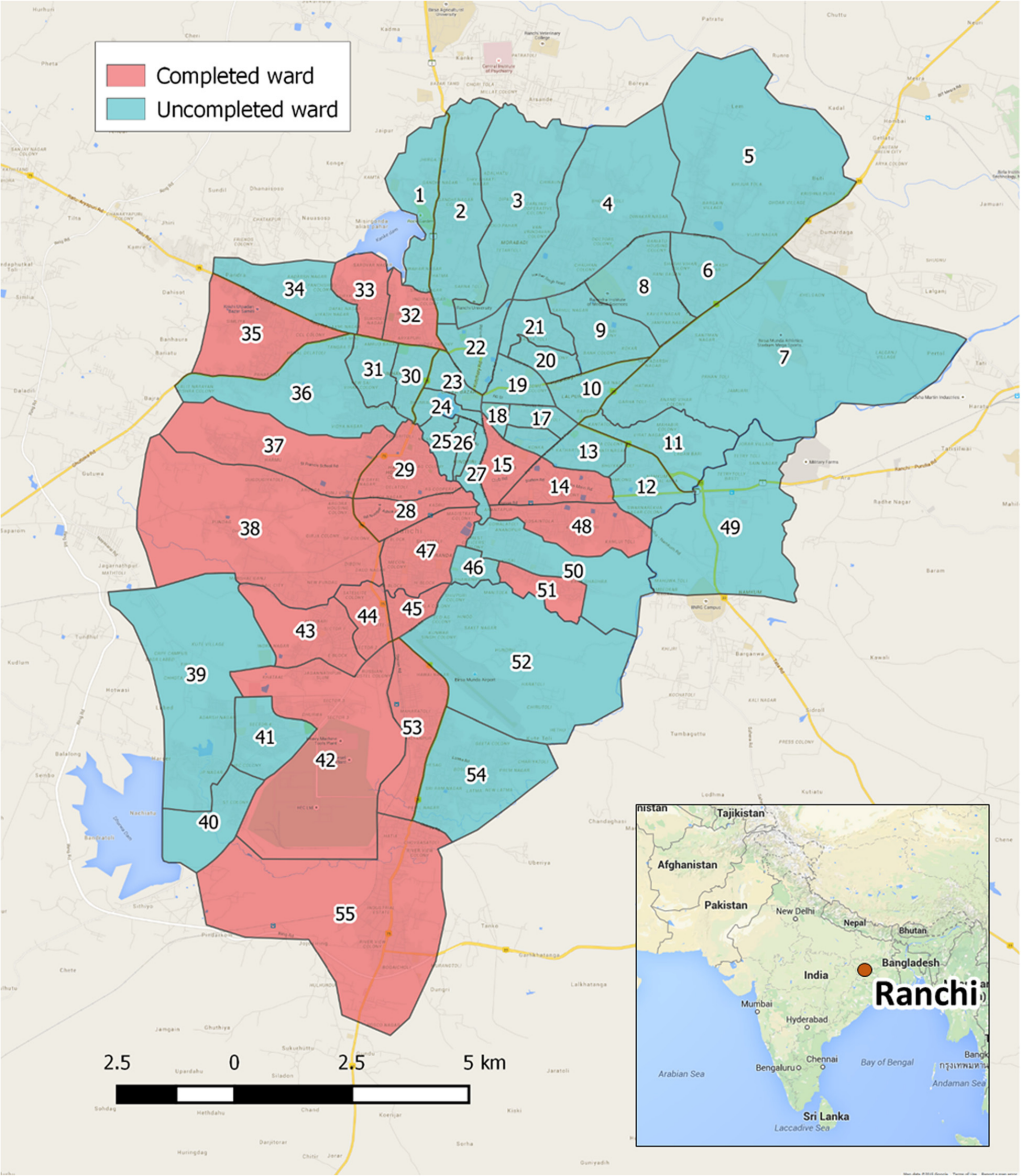
Map of Ranchi showing ward boundaries and area of study, reproduced manually in QGIS from Ranchi Municipal Corporation ward map. Map data ©2015 Google Maps

Ranchi (23°22′N, 85°20′E) is the capital city of the North East Indian state of Jharkhand, with an urban human population of 1.07 million people [14]. The region has a humid subtropical climate with highest rainfall between June and September. The city is divided into 55 administrative wards (Fig. 1) and has a large free roaming dog population. Mission Rabies works in partnership with the local non-governmental organisation HOPE & Animal Trust which was established in 2000 in response to the perceived high level of suffering seen in the free roaming dog population and the lack of local veterinary services accessible to these animals. HOPE & Animal Trust focuses on sterilization of dogs and cats, and rehabilitation and rehoming of animals that cannot be safely return to their point of capture. HOPE & Animal Trust have a Memorandum of Understanding with Ranchi Municipal Corporation to conduct mass rabies vaccination and sterilization of dogs within Ranchi Municipality. Dogs included in the study were those vaccinated, marked and released (VMR) by roaming vaccination teams and those sterilized as a part of the catch-neuter-vaccinate-return (CNVR) programme which ran in parallel to the rotating vaccination work. The study period was from 5^th^ December 2014 to 16^th^ April 2015. During this period 94 days were spent administering rabies vaccinations.

### Mission Rabies App

A bespoke ‘Mission Rabies’ App was developed which enabled information about each dog vaccinated to be recorded on a smartphone at the time of their vaccination. This information was then synchronized via WiFi or 3G to a web based server once an internet connection was available. The dataset for each dog vaccinated included GPS location, manually entered ward number, action taken (vaccinated, marked and released/vaccinated and released but not marked/previously vaccinated within 1 year, marked/taken to clinic), sex (male/female), ownership status (presented by owner/free roaming), approximate age (< 3 months, > 3 months), neuter status defined by presence/absence of an ear notch routinely performed at the time of surgery (neutered/not-neutered), body condition score (BCS: emaciated (1), underweight (2), healthy weight (3), obese (4)) [15], presence of alopecia (four point score of alopecia affecting a percentage of total body surface area; normal (no hair loss), mild (< 20 % hair loss), moderate (20–80 % hair loss), severe (> 80 % hair loss)), other disease (transmissible venereal tumour, wounds, lameness, other). Ward boundaries were displayed on the app to enable teams to navigate through the ward and stay within boundaries. Three Samsung Galaxy Core 2 phones were used, one with each vaccination team and one with the surveyor. These belonged to the charity and were budgeted for in the cost of the vaccination campaign.

### Vaccinate-assess-move protocol

Vaccination teams consisted of one vet, two assistants and four dog catchers/handlers. For the majority of the time one vaccination team was operating, with a second team working intermittently depending on staff availability. Vaccines were carried in cool boxes containing ice packs wrapped in newspaper to avoid direct contact and freezing of vaccine. The project manager allocated the vaccination teams a ward within which to work each day. The teams would walk through every street in the ward, catching dogs of all ages not already identified as vaccinated. Dogs that could be handled were restrained manually for vaccination, whilst dogs that could not be approached and were caught and restrained using light-weight aluminium framed butterfly nets. Once restrained dogs were vaccinated intramuscularly or subcutaneously (Nobivac® Rabies, MSD Animal Health), marked and released. Vaccinated dogs were marked with non-toxic paint along the top and back of the head to allow for identification on post-vaccination surveys and prevent repeat vaccination [16]. Vaccination teams continued within the same ward on consecutive days until the vaccination team reported that the maximum number of dogs had been vaccinated, at which time the project manager was informed and a post vaccination survey was undertaken.

### Post-vaccination survey protocol

**Fig. 2:**
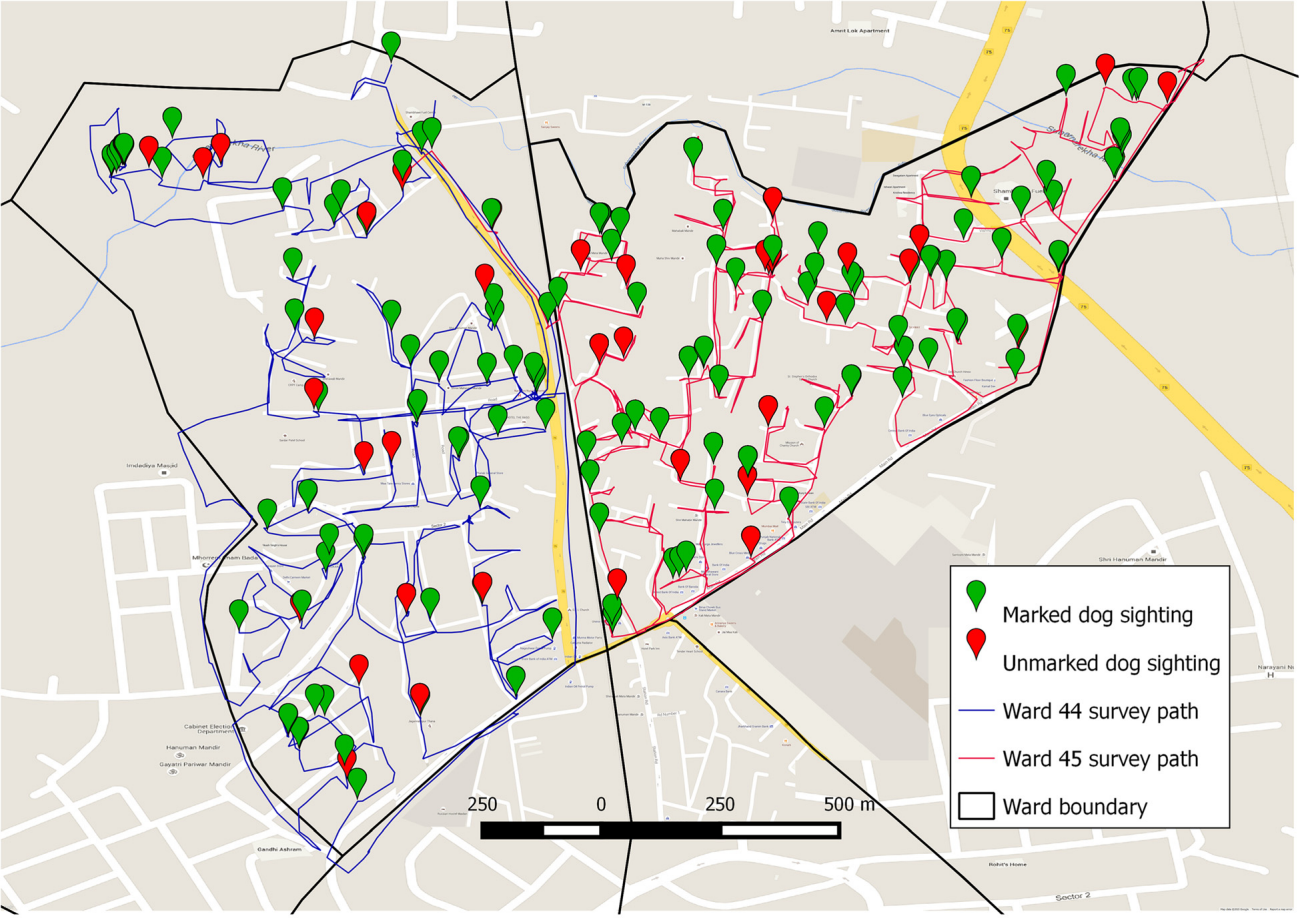
Map showing a representative subset of dog sightings and survey paths for surveys of Ward 44 and Ward 45. Map data ©2015 Google Maps

Following completion of the initial cycle of vaccinations in each ward, a surveyor travelled around the ward by motorbike, navigating using the smartphone map to cover every street within the ward boundaries. Surveys were conducted in the morning between first light and 11 am and in the late afternoon between 3 pm and dusk. Only free roaming dogs were recorded, therefore dogs tied or confined to private property were not recorded. Each dog sighted was entered into a ‘Survey form’ on the ‘Mission Rabies’ App which included GPS location, sex and age (adult male/adult non-lactating female/adult lactating female/puppy), neuter status (ear notch present/absent), vaccination status (mark present/absent). The data was synchronized to the central server via WiFi or 3G as described above. Incorporated into the app was a ‘path tracker’ function which recorded the path travelled during the survey. If more than 70 % of sighted dogs were marked the ward was considered complete, whereas if coverage was less than 70 %, vaccination teams were directed back to the ward to vaccinate unmarked dogs and the survey to assess vaccination coverage was subsequently repeated (Fig. 2).

### Data analysis

For project management purposes data summaries and maps can be viewed in real time on the ‘Mission Rabies’ app backend. Calculations of vaccination coverage were undertaken in Excel 2013 (Microsoft Inc., Redmond, WA). For more detailed analysis both vaccination and survey datasets were downloaded from the server as CSV files. Ward boundaries were imported into ArcGIS Desktop 10.3 and dog sighting (survey) and vaccination locations were labelled with the Ward according to GPS location. Data was then exported into Excel 2013 cleaning and analysis. Maps were prepared for presentation in QGIS Desktop 2.6.1 (QGIS development team, Open Source Geospatial Foundation Project). Vaccination coverage was estimated as proportion of sighted dogs which were marked with associated exact binomial confidence interval. Data analysis was performed using the R statistical system.

## Results

**Fig. 3:**
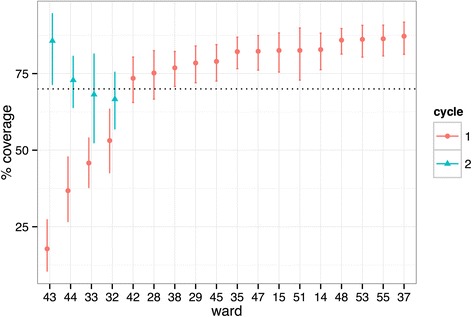
Plot showing coverage of ward after first cycle of vaccination (red circles) and after second cycle of vaccination (blue triangle). Mean vaccination coverage is shown by either the circle or triangle with the 2.5-97.5 confidence intervals shown by the vertical line. The dotted line represents 70 % vaccination coverage

The study protocol was developed during the vaccination of dogs in 13 wards at the start of the project. This study reports the vaccination numbers and coverage during the subsequent 18 wards Additional file 1. The app was taken offline for a six day period in December 2014 and a three day period in February 2015 for programming upgrades. During this time data which involved 3 wards was not available for inclusion in this study. In the remaining 21 wards, the vaccination programme is currently ongoing. The number of owned and free roaming dogs vaccinated is shown in Table 1. The percentage of dogs vaccinated was assessed after the initial vaccination cycle. The percentage vaccine coverage is shown in Table 1 alongside 2.5 and 97.5 confidence intervals in Fig. 3. In 14 of the 18 wards, vaccination coverage was found to be over 70 % after the initial round of vaccinations.

**Table 1:** Number of owned and free roaming dogs vaccinated, and the percentage vaccination coverage achieved, during the initial vaccination cycle

Ward	Vaccination		Primary survey		
	No. of owned dogs vaccinated	No. of free roaming dogs vaccinated	Total dogs sighted	No. of marked resighted	% coverage
14	53	306	169	140	82.8
15	48	313	149	123	82.6
28	6	197	125	94	75.2
29	13	447	195	153	78.5
32	3	175	96	51	53.1
33	6	296	155	71	45.8
35	42	223	230	189	82.2
37	39	368	172	150	87.2
38	42	383	225	173	76.9
42	47	580	147	108	73.5
43	10	267	90	16	17.8
44	19	278	87	32	36.8
45	91	246	195	154	79.0
47	33	312	192	158	82.3
48	60	441	291	250	85.9
51	16	154	86	71	82.6
53	23	329	188	162	86.2
55	88	639	198	171	86.4
Total	639	5954	3140	2267

In four wards which had a vaccination coverage of below 70 %, a second cycle of vaccinations was performed. This resulted in the vaccination of an additional 311 dogs. Following this second cycle of vaccinations, a second assessment of vaccination coverage was performed. This demonstrated that two of the four wards had a vaccination coverage of over 70 % with the two remaining wards having a vaccination coverage of just below 70 %, namely 66.7 % and 68.2 % (Table 2, Fig. 3).

**Table 2:** Number of owned and free roaming dogs vaccinated, and the percentage vaccination coverage achieved, during the initial vaccination cycle

		Second Vaccination Cycle		Second Survey		
Ward	% coverage during first vaccination cycle	Additional owned dogs vacc'd in 2nd cycle	No. of free roaming dogs vacc'd in 2nd cycle	No. of total dogs observed in 2nd resighting	No. of marked dogs in 2nd resighting	% coverage
32	53.1	4	77	108	72	66.7
33	45.8	2	85	44	30	68.2
43	17.8	4	68	42	36	85.7
44	36.8	2	69	118	86	72.9
Total		12	299	312	224	

**Fig. 4:**
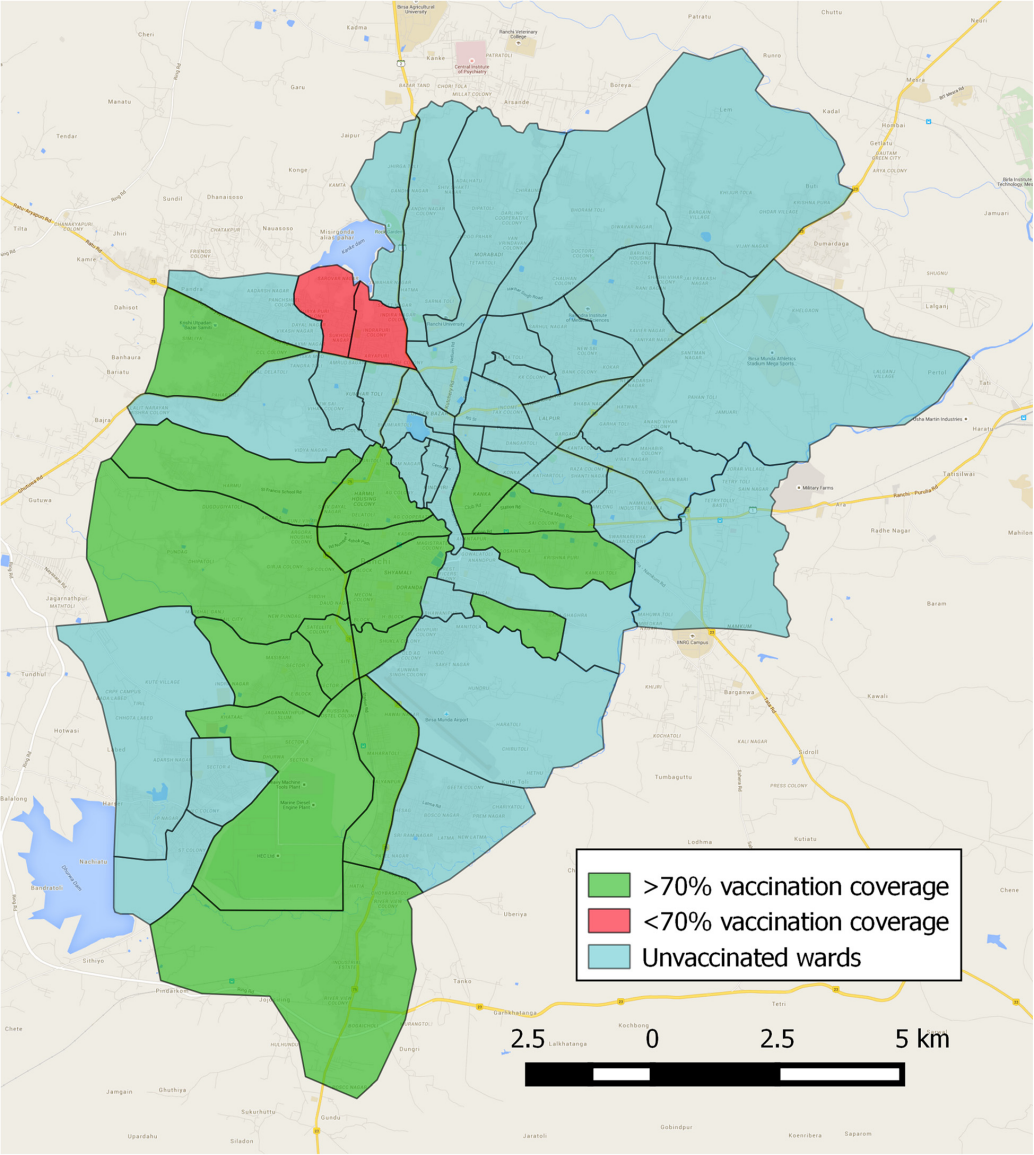
Map of Ranchi showing mean vaccination coverage by ward following the second cycle of vaccinations. Map data ©2015 Google Maps

In summary, this approach ensured that after the second round of vaccinations, 6904 dogs were vaccinated and in 16 of the 18 wards, a vaccination coverage of over 70 % had been achieved. Importantly, not only was the mean vaccination coverage over 70 % in 16 of the 18 wards following the second cycle of vaccinations (Fig. 4), but the 2.5 % lower confidence interval was above 70 % in 13 of the 18 wards.

The number of free roaming and owned dogs which entered either the capture, neuter, vaccinate and release (CNVR) and vaccinate, mark and release (VMR) programme is shown in Table 3. The majority (88.4 %) of dogs within the VMR population were free roaming of which 65.6 % were male. This compares to 83.7 % of dogs presented by an owner for vaccination which were male.

**Table 3:** Number of free roaming and owned dogs vaccinated through the capture, neuter, vaccinate and release (CNVR) and vaccinate, mark and release (VMR) programme

Confinement	CNVR	VMR	Total
Free Roaming	1441	4812	6253
Presented by owner	20	631	651
Total	1461	5443	6904

The neuter status of the free roaming and owned dogs is shown in Table 4. 76.1 % of the free roaming VMR population were neutered, compared to 10.6 % of the dog population presented by owners (Table 4).

**Table 4:** Neuter status within VMR population for owned and free roaming dogs

Neuter status	Free Roaming	Presented by owner	Total
Entire	1216	568	1784
Neutered	3595	63	3658
Unknown	1		1
Total	4812	631	5443

Skin disease was reported in 1.9 % of free roaming dogs in the VMR population and 16.6 % were reported as underweight or emaciated (BCS 1 or 2).

## Discussion

India has the highest number of human deaths from rabies of any country [1, 17]. The administration of canine rabies vaccines to more than 70 % of dogs has been well documented in numerous countries to greatly reduce the incidence of rabies in both human and canine populations [13, 18–20]. Despite the pressing need for effective canine rabies vaccination programmes in India, there have been few publications describing canine rabies vaccination strategies in the country with the highest rabies burden [8, 9, 11]. Specifically, there is a paucity of publications which have demonstrated that a large number of dogs can be vaccinated in a short period of time at a high vaccination coverage. For example, a recent study in India reported the vaccination of an average of 47 dogs (range 22–69) in six Indian villages at a median vaccination coverage of 34 % [10]. The authors highlighted the challenges of handling dogs and misconceptions regarding dog vaccination as barriers to achieving a higher vaccination coverage. Another study reported a vaccination coverage of 35.5 % although a larger number of dogs were vaccinated during this long term project [21]. Consequently, our programme, which vaccinated over 6900 dogs in 94 days and achieved a mean vaccination coverage of over 70 % in 16 of the 18 wards robustly demonstrates, for the first time, that large number of free roaming dogs can be vaccinated at high coverage in India in a short period of time.

An important outcome of our study is not only that almost all wards had a mean vaccination coverage of over 70 %, but that the lowest confidence interval for vaccine coverage was over 70 % in nearly three quarters of all the wards. This demonstrates that for the vast majority of wards, we can be confident that high vaccine coverage was achieved. Furthermore, our approach demonstrated the value of examining vaccine coverage in smaller areas within large cities. For example, vaccine coverage across a large city may be over 70 % yet there might be large enough populations of dogs in areas of low vaccine coverage which could allow rabies to be maintained within the dog population. By examining coverage in numerous small wards throughout the city, we have been able to demonstrate that the lower vaccination coverage confidence value was never below 52 % and in 16 of the 18 wards was above 63 %.

In this study, the use of mobile technology and a tailor made smartphone app to provide specific functionalities of data entry and boundaries displayed on maps, enabled efficient and simple region wise direction of catching and survey teams. Although mobile phone technology has been used in other epidemiology studies [22], this is the first study to report the development and implementation of a bespoke app tailored towards the collection of data relevant in canine rabies field work. Data captured remotely in the field was tagged with GPS location and synchronized to a web based server. The project manager could then instantly view maps of where the vaccination teams had been working or download data in spreadsheet format for estimation of vaccination coverage enabling the prompt direction of teams back to areas with low coverage. This instant access to digitalised data saved significant management time in conversion of paper records into an electronic spreadsheet and therefore made the application of the system more appealing and sustainable at the project management level. The resulting dataset also facilitates study of dog demographics and spatial analysis. Designation of boundaries on Google maps enables a complex geographic area with poor existing mapping, defined regions or road names such as Ranchi to be systematically searched for the presence of dogs. This technology was crucial in allowing the field teams to vaccinate a high percentage of the dog population. Assessing the cost-benefit of using mobile technology over other paper records are outside of the scope of this study, however basic smart phones are becoming increasingly affordable and when factored into the overall budget for mass vaccination campaigns, the cost of using smart phones per dog vaccinated is minimal. The benefits in improved reporting, team direction, impact assessment and project management have been found to be invaluable in the authors’ experience managing multiple remote projects on a large scale.

Our study describes a vaccination programme which successfully addressed the dual challenges of vaccinating a large number of dogs at a high vaccination coverage in a relatively short period of time. There is often a culture of quasi-ownership in India whereby members of the community feed free roaming dogs, and therefore support their survival and reproduction, however, little responsibility is taken to ensure that the dog can be handled or that rabies vaccination or sterilization occurs. This produces a profound public health and animal welfare risk and means that accessing 70 % of the free roaming dog population for vaccination is more challenging. A major challenge in Ranchi, which is typical of many India cities, was the high proportion of dogs which were free roaming (92 %). Unlike in Africa, where a large number of dogs are owned and can be vaccinated through static point vaccine approaches [13, 18, 23], the vast majority of dogs in Ranchi were not identifiably owned. In this study teams used butterfly nets to catch and restrain the large number of dogs which were not amenable to handling. The use of butterfly nets has been previously described in vaccination campaigns in Bali where the majority of dogs were not amenable to handling [7, 24] and has been found to be more effective and humane than other capture methods in this situation, however the approach and methods must be tailored to each local setting. Minimising the detrimental impact on the welfare of each animal treated whilst achieving the greatest possible benefit to the wider human and animal populations through rabies control need to be carefully balanced and continuously reviewed and refined.

The approach of assessing vaccination coverage by recording the number of marked, vaccinated and unmarked, unvaccinated dogs has been widely reported. This simple method of marking vaccinated dogs followed by dog-sight surveys to estimate vaccination coverage in the abundant free roaming dog populations is a cheap and effective system to estimate vaccination coverage in real-time. Mass vaccination campaigns provide an ideal opportunity for gathering information about a large cross section of the population with minimal additional effort which can then be used to better direct resources and refine effective methods [10, 25]. Measurement of demographic data such as body condition score, skin condition and reproductive status enables monitoring of change in the population over sequential vaccination campaigns and assessment of the impact of other interventions such as sterilization and education activities. The sex distribution observed in this study is comparable to previous reports on Indian dog demographics [26–28].

The problem of dogs in India is often perceived to be one of “too many dogs on the street” as opposed to rabies being the prime problem, with the latter being a far easier issue to address in the short term if handled in isolation. The problem of free roaming dog over population is more complex, with cultural and ecological root causes which take longer to influence than dog vaccination alone. Long-term, sustained CNVR programmes in Rajasthan have successfully eliminated rabies as well as reducing dog population turnover, therefore enhancing persistence of vaccinated animals in the community [15, 21, 29]. A study in Jaipur reported sterilization of 66 % of the female dog population resulted in a 28 % reduction in roaming dogs over the eight year period of work [21]. In this study 78 % of the roaming dog population was estimated to be sterilize, which is likely to have resulted in a similar reduction in population turnover. Given the sheer size of the dog population in many Indian cities, it is unlikely to be cost-effective, logistically feasible or ecologically beneficial to conduct blanket CNVR interventions with the aim of controlling rabies nationally. Instead dog population management may be viewed as a separate undertaking which is based on targeting dogs most likely to contribute to the problem on a location-by-location basis. VMR does not require the same level of veterinary expertise, infrastructure, equipment and consumable costs that are needed in CNVR which also requires additional investment in monitoring and quality control to ensure animal welfare is upheld to the highest possible standards. Therefore, more emphasis should be placed on mass dog vaccination in order to reduce the incidence of rabies over a large area in the shorter term [30]. Further study is needed to assess the cost effectiveness of mass canine vaccination initiatives such as the one developed in this study.

## Conclusions

In summary, this study describes a vaccination programme which has allowed rabies vaccines to be administered to over 6900 dogs in India in 94 days with a mean vaccination coverage of over 70 % in 16 of the 18 studied wards. Our study demonstrates the feasibility of vaccinating large number of dogs at high coverage in India even when the vast majority of dogs are free roaming. If our approach is rolled out more extensively across India, this vaccination strategy has the potential to significantly reduce the incidence of rabies in both dog and human populations.

## Ethics approval

Ethical approval for this research has been granted by University of Edinburgh’s Veterinary Ethics Research Committee (64/15: Investigation of rabies vaccination approaches).

## Availability of data and materials

The vaccination and survey datasets containing individual dog vaccination and dog sight survey data are available in supporting documents.

## Additional file

Additional file 1: Complete dataset of dogs vaccinated during study. (XLSX 1444 kb)

## Abbreviations

CNVR: Catch neuter vaccinate release
VMR: vaccinate mark release
